# Higher Postoperative Mortality and Inferior Survival After Right-Sided Liver Resection for Perihilar Cholangiocarcinoma: Left-Sided Resection is Preferred When Possible

**DOI:** 10.1245/s10434-024-15115-0

**Published:** 2024-03-12

**Authors:** Pim B. Olthof, Joris I. Erdmann, Ruslan Alikhanov, Ramón Charco, Alfredo Guglielmi, Jeroen Hagendoorn, Abdul Hakeem, Frederik J. H. Hoogwater, William R. Jarnagin, Geert Kazemier, Hauke Lang, Shishir K. Maithel, Massimo Malago, Hassan Z. Malik, Silvio Nadalin, Ulf Neumann, Steven W. M. Olde Damink, Johann Pratschke, Francesca Ratti, Matteo Ravaioli, Keith J. Roberts, Erik Schadde, Andreas A. Schnitzbauer, Ernesto Sparrelid, Baki Topal, Roberto I. Troisi, Bas Groot Koerkamp, Perihilar Cholangiocarcinoma Collaboration Group, L. Aldrighetti, F. Bartsch, W. O. Bechstein, J. Bednarsch, C. M. A. de BenzingBoer, S. A. Bouwense, I. Capobianco, M. Cescon, M. I. D’Angelica, M. Dewulf, P. de Reuver, E. de Savornin Lohman, M. Efanov, L. C. Franken, J. Geers, M. C. Giglio, S. Gilg, C. Gomez-Gavara, T. M. van Gulik, J. Heil, J. N. M. IJzermans, H. Jansson, T. P. Kingham, P. Lodge, R. Margies, R. Marino, Q. I. Molenaar, T. A. Nguyen, L. E. Nooijen, C. L. M. Nota, E. Poletto, R. J. Porte, R. Prasad, L. M. Quinn, J. Rolinger, A. Ruzzenente, M. Schmelzle, M. Serenari, A. Sultana, S. van Laarhoven, B. M. Zonderhuis

**Affiliations:** 1 Department of Surgery, Erasmus Medical Center, Rotterdam, The Netherlands; 2 Department of Surgery, Amsterdam UMC, Amsterdam, The Netherlands; 3 Department of Surgery, University Medical Center, Groningen, Groningen The Netherlands; 4 Department of Liver and Pancreatic Surgery, Department of Transplantation, Moscow Clinical Scientific Centre, Moscow, Russia; 5 Department of HBP Surgery and Transplantation, Hospital Universitario Vall d’Hebron, Universidad Autónoma de Barcelona, Barcelona, Spain; 6 Division of General Surgery, Department of Surgery, Unit of Hepato-Pancreato-Biliary Surgery, University of Verona Medical School, Verona, Italy; 7 Department of Surgical Oncology, University Medical Centre/Utrecht University, Utrecht, The Netherlands; 8 Division of Surgery, Department of Hepatobiliary and Liver Transplant Surgery, St James’s University Hospital, Leeds, UK; 9 Hepatopancreatobiliary Service, Department of Surgery, Memorial Sloan Kettering Cancer Center, New York, NY USA; 10 Department of General, Visceral and Transplantation Surgery, University Medical Center, Mainz, Germany; 11 Division of Surgical Oncology, Winship Cancer Institute, Emory University, Atlanta, GA USA; 12 Department of HPB and Liver Transplantation Surgery, University College London, Royal Free Hospitals, London, UK; 13 Aintree University Hospital, Liverpool, UK; 14 Department of General and Transplant Surgery, University Hospital Tübingen, Tübingen, Germany; 15 Department of Surgery and Transplantation, University Hospital RWTH Aachen, Aachen, Germany; 16 Department of Surgery, Maastricht University Medical Center (MUMC), Maastricht, The Netherlands; 17 Department of Surgery, Campus Charité Mitte and Campus Virchow-KlinikumCharité-Universitätsmedizin Berlin, Berlin, Germany; 18 Hepatobiliary Surgery Division, IRCCS San Raffaele Scientific Institute and Vita-Salute San Raffaele University, Milan, Italy; 19 General Surgery and Transplant Unit, IRCCS Azienda Ospedaliero-Universitaria di Bologna, Bologna, Italy; 20 Department of Surgery, University Hospital Birmingham, Birmingham, UK; 21 Department of Surgery, Rush University Medical Center Chicago, Chicago, IL USA; 22 Universitätsklinikum Frankfurt, Klinik für AllgemeinViszeral und Transplantationschirurgie, Frankfurt, Germany; 23 Division of Surgery and Oncology, Karolinska Institutet, Karolinska University Hospital, Stockholm, Sweden; 24 Department of Surgery, Catholic University of Leuven, Leuven, Belgium; 25 Department of Clinical Medicine and Surgery, Division of HBP, Minimally Invasive and Robotic Surgery, Transplantation Service, Federico II University Hospital, Naples, Italy

## Abstract

**Background:**

A right- or left-sided liver resection can be considered in about half of patients with perihilar cholangiocarcinoma (pCCA), depending on tumor location and vascular involvement. This study compared postoperative mortality and long-term survival of right- versus left-sided liver resections for pCCA.

**Methods:**

Patients who underwent major liver resection for pCCA at 25 Western centers were stratified according to the type of hepatectomy—left, extended left, right, and extended right. The primary outcomes were 90-day mortality and overall survival (OS).

**Results:**

Between 2000 and 2022, 1701 patients underwent major liver resection for pCCA. The 90-day mortality was 9% after left-sided and 18% after right-sided liver resection (*p* < 0.001). The 90-day mortality rates were 8% (44/540) after left, 11% (29/276) after extended left, 17% (51/309) after right, and 19% (108/576) after extended right hepatectomy (*p* < 0.001). Median OS was 30 months (95% confidence interval [CI] 27–34) after left and 23 months (95% CI 20–25) after right liver resection (*p* < 0.001), and 33 months (95% CI 28–38), 27 months (95% CI 23–32), 25 months (95% CI 21–30), and 21 months (95% CI 18–24) after left, extended left, right, and extended right hepatectomy, respectively (*p* < 0.001). A left-sided resection was an independent favorable prognostic factor for both 90-day mortality and OS compared with right-sided resection, with similar results after excluding 90-day fatalities.

**Conclusions:**

A left or extended left hepatectomy is associated with a lower 90-day mortality and superior OS compared with an (extended) right hepatectomy for pCCA. When both a left and right liver resection are feasible, a left-sided liver resection is preferred.

**Supplementary Information:**

The online version contains supplementary material available at 10.1245/s10434-024-15115-0.

For patients diagnosed with perihilar cholangiocarcinoma (pCCA), complete surgical resection of the tumor is the only treatment that offers a chance for long-term survival. Only a minority of about 15% of patients with pCCA can undergo surgical resection.^1^ Reported 5-year survival rates after resection range from 21 to 35%.^2,3^ With palliative chemotherapy, the median overall survival (OS) is 12 months, and only 3 months without treatment.^1,4,5^

Complete resection of pCCA usually requires an extrahepatic bile duct resection combined with major hepatectomy. These extensive surgical procedures are associated with high risks of liver failure, with a postoperative 90-day mortality rate of 12% in a recent systematic review.^6^ Postoperative mortality is mostly explained by postoperative liver failure.^7^ Numerous small single-center studies have compared the outcomes for patients with pCCA after left and right hepatectomy.^8,9^ In some patients, unilateral liver atrophy or portal vein involvement dictate the side of liver resection. In other patients, both a left- and right-sided resection can aim for a complete (i.e., R0) resection. The advantage of a right-sided liver resection is the long left hepatic ductal margin.^10^ However, a right-sided liver resection is associated with a higher risk of postoperative mortality, because of a smaller liver remnant, compared with left-sided resections.^11,12^ It remains uncertain whether the added oncological benefit of a wider margin outweighs the increased surgical risk of a right-sided resection.

This study aimed to compare 90-day postoperative mortality and long-term OS between right- and left-sided major liver resections in the pCCA collaboration group.

## Methods

All consecutive patients aged 18 years or older who underwent resection of proven or suspected pCCA at 25 European and American centers, during various periods after 2000, were included in the collaborative database. Data were collected through a standardized and pseudo-anonymous data file. The definition of pCCA was a biliary tumor originating between the segmental bile ducts and cystic duct. The Institutional Medical Ethics Committee of Erasmus MC waived the need for ethical approval. The study was conducted in adherence with the Strengthening the Reporting of Observational Studies in Epidemiology (STROBE) guidelines.

The work-up and management of patients included in the study differed across the centers and during the period of inclusion. Major liver resection was defined as resection of at least three Couinaud liver segments;^13^ left hepatectomy was defined as the resection of liver segments 2, 3, and 4, with or without segment 1; right hepatectomy was defined as the resection of liver segments 5, 6, 7, and 8, with or without segment 1; and an extended liver resection was defined as any extension beyond a formal left or right hepatectomy. Left-sided resections include both left and extended left hepatectomy, and right-sided resections include both right and extended right hepatectomy. Preoperative cholangitis was defined as fever and leukocytosis requiring (additional) biliary drainage in accordance with the definitions applied in the DRAINAGE trial.^14^ Tumors were classified according to the Bismuth–Corlette classification. Negative resection margins were defined as tumor-free margins in all resection margins reported in the pathology report. All complications within 30 days after surgery or during initial hospitalization were scored and classified according to the classification proposed by Dindo et al. ^15^ Postoperative mortality was defined as mortality within 90 days after surgery. Liver failure, biliary leakage, and hemorrhage were scored and graded according to the respective International Study Group of Liver Surgery (ISGLS) criteria, and only grades B and C were considered clinically relevant.^16–18^ OS was defined as the time between surgery and death or last follow-up.

All categorical variables were shown as numbers with percentages and differences were tested using the Chi-square or Fisher’s exact test where appropriate. Continuous variables were shown as medians with interquartile ranges (IQR) and differences were tested using Kruskal–Wallis tests. Survival rates and hazard ratios were shown as medians with 95% confidence intervals (CIs). Survival curves were generated using the Kaplan–Meier method and differences between groups were tested using log-rank tests. Median follow-up was calculated using the reverse Kaplan–Meier method. Univariable and multivariable analysis to identify factors associated with 90-day mortality were performed using binary logistic regression analysis, and the analyses for OS were performed using Cox regression analysis in accordance with the TRIPOD checklist. All variables with a *p*-value <0.1 at univariable analysis were included in the multivariable analysis. All statistical analyses were performed using SPSS version 26 (IBM Corporation, Armonk, NY, USA) and figures were generated using GraphPad version 9 (GraphPad, La Jolla, CA, USA).

## Results

A total of 2065 patients underwent resection for proven or suspected pCCA across 25 centers. Patients who had a postoperative diagnosis other than pCCA (*n* = 178) were excluded, as were patients who only underwent an extrahepatic bile duct resection without liver resection (*n* = 146) and patients who underwent minor liver resection (*n* = 40). The remaining 1701 patients were included in the analyses, with a median center volume of 62 (33–79).

Clinicopathological characteristics as well as outcomes of patients according to the type of hepatectomy performed are shown in Table 1. Extended right hepatectomy was the most frequent resection (*n* = 576, 34%), followed by left hepatectomy (*n* = 540, 32%), right hepatectomy (*n* = 309, 18%), and extended left hepatectomy (*n* = 276, 16%). Patients who underwent an extended right hepatectomy were younger compared with patients undergoing the other types of resection (62 vs. 66 years; *p* < 0.001), and more frequently underwent preoperative biliary drainage (89% vs. 79%; *p* < 0.001). Portal vein reconstruction was performed in 45% of patients who underwent an extended hepatectomy, compared with 18% with a formal right or left hepatectomy (*p* < 0.001). Nodal status and resection margin were similar across the different types of hepatectomy.

**Table 1:** Patient and disease characteristics as well as outcomes according to the type of hepatectomy performed for perihilar cholangiocarcinoma

		Type of hepatectomy performed					
	All	Left [*n* = 540]	Extended left [*n *= 276]	Right [*n* = 309]	Extended right [*n* = 576]	*p*-Value	
Age, years [median (IQR)]	65 (57–72)	66 (57–72)	66 (57–73)	67 (59–73)	62 (54–70)	<0.001	
Male sex	981 (58)	332 (61)	154 (56)	193 (62)	302 (52)	0.003	
ASA score III/IV	545 (32)	181 (34)	101 (37)	96 (31)	167 (29)	0.110	
Bismuth classification						<0.001	
I/II	244 (14)	82 (15)	17 (6)	75 (24)	70 (12)		
IIIA	540 (32)	38 (7)	49 (18)	159 (52)	294 (52)		
IIIB	448 (26)	312 (59)	99 (37)	12 (4)	25 (4)		
IV	440 (26)	100 (19)	106 (39)	61 (20)	173 (31)		
Preoperative biliary drainage						<0.001	
None	295 (17)	133 (25)	45 (16)	56 (18)	58 (11)		
PTBD	412 (24)	145 (27)	63 (23)	94 (30)	110 (19)		
EBD	749 (44)	197 (36)	137 (50)	127 (41)	288 (50)		
Both	248 (15)	65 (12)	31 (11)	32 (10)	120 (21)		
Preoperative cholangitis	342 (20)	111 (21)	44 (16)	73 (24)	114 (20)	0.139	
Portal vein embolization	331 (19)	19 (4)	4 (1)	61 (20)	247 (43)	<0.001	
Portal vein reconstruction	535 (31)	90 (17)	105 (38)	62 (20)	278 (48)	<0.001	
Node positive (pN+)	717 (42)	217 (40)	122 (44)	132 (43)	246 (43)	0.703	
Metastatic disease	74 (4)	21 (4)	11 (4)	8 (3)	34 (6)	0.117	
Negative margin	1109 (65)	365 (68)	169 (61)	209 (68)	376 (65)	0.265	
Poor differentiation	390 (23)	101 (19)	79 (29)	75 (24)	135 (23)	0.012	
Perineural invasion	1133 (66)	348 (64)	174 (63)	199 (64)	412 (71)	0.177	
Major morbidity	831 (49)	208 (39)	126 (46)	162 (52)	335 (58)	<0.001	
Liver failure, ISGLS B/C	274 (16)	48 (9)	32 (12)	58 (19)	136 (23)	<0.001	
Biliary Leakage, ISGLS B/C	339 (20)	103 (19)	64 (23)	59 (19)	113 (19)	0.516	
Hemorrhage, ISGLS B/C	112 (7)	28 (5)	19 (7)	22 (7)	43 (7)	0.462	
30-day mortality	149 (9)	31 (6)	16 (6)	32 (10)	70 (12)	<0.001	
90-day mortality	232 (14)	44 (8)	29 (11)	51 (17)	108 (19)	<0.001	

Data are expressed as *n* (%) unless otherwise specified

*ASA* American society of anesthesiologists, *PTBD* Percutaneous transhepatic biliary drainage, *EBD* Endoscopic biliary drainage, *ISGLS* International study group of liver surgery, *IQR* Interquartile range

The 90-day mortality was 9% after left-sided liver resection and 18% after right-sided liver resection (*p* < 0.001). The 90-day mortality rates were 8% (44/540) after left, 11% (29/276) after extended left, 17% (51/309) after right, and 19% (108/576) after extended right hepatectomy (*p* < 0.001). Similarly, post-hepatectomy liver failure rates were 9% (48/309), 12% (32/276), 19% (58/309), and 24% (136/576) after left, extended left, right, and extended right hepatectomy, respectively (*p* < 0.001).

Independent risk factors for 90-day mortality were right (odds ratio [OR] 2.17, 95% CI 1.30–3.60) and extended right liver resection (OR 2.59, 95% CI 1.63–4.14) when compared with left liver resection (Table 2). Other independent risk factors were age (OR 1.03, 95% CI 1.02–1.05), American Society of Anesthesiologists (ASA) III/IV (OR 1.44, 95% CI 1.05–1.97), and preoperative cholangitis (OR 1.48, 95% CI 1.04–2.11).

**Table 2:** Univariable and multivariable analysis for 90-day mortality after surgery for perihilar cholangiocarcinoma

	Univariable		Multivariable	
	OR (95% CI)	*p*-Value	OR (95% CI)	*p*-Value
Age	1.03 (1.02–1.05)	<0.001	1.03 (1.02–1.05)	<0.001
Male sex	1.19 (0.89–1.57)	0.239		
ASA score III/IV vs. I/II	1.59 (1.19–2.13)	0.002	1.44 (1.05–1.97)	0.022
Bismuth type				
I/II	Reference		Reference	
IIIA	1.02 (0.67–1.55)	0.928	0.78 (0.50–1.21)	0.265
IIIB	0.59 (0.37–0.94)	0.026	0.88 (0.52–1.51)	0.648
IV	0.98 (0.63–1.51)	0.916	0.89 (0.56–1.42)	0.617
Tumor size, >3 cm	1.35 (0.99–1.85)	0.061	1.29 (0.95–1.76)	0.101
Biliary drainage	1.39 (0.93–2.07)	0.112	1.13 (0.71–1.80)	0.614
Preoperative cholangitis	1.44 (1.04–2.00)	0.030	1.48 (1.04–2.11)	0.031
Type of hepatectomy				
Left	Reference		Reference	
Extended left	1.32 (0.81–2.16)	0.269	1.16 (0.69–1.95)	0.580
Right	2.22 (1.45–3.42)	<0.001	2.17 (1.30–3.60)	0.003
Extended right	2.61 (1.80–3.79)	<0.001	2.59 (1.63–4.14)	<0.001
Portal vein resection	1.50 (1.13–2.00)	0.005	1.35 (0.98–1.85)	0.066

*ASA* American society of anesthesiologists, *CI* Confidence interval, *OR* Odds ratio

**Fig. 1:**
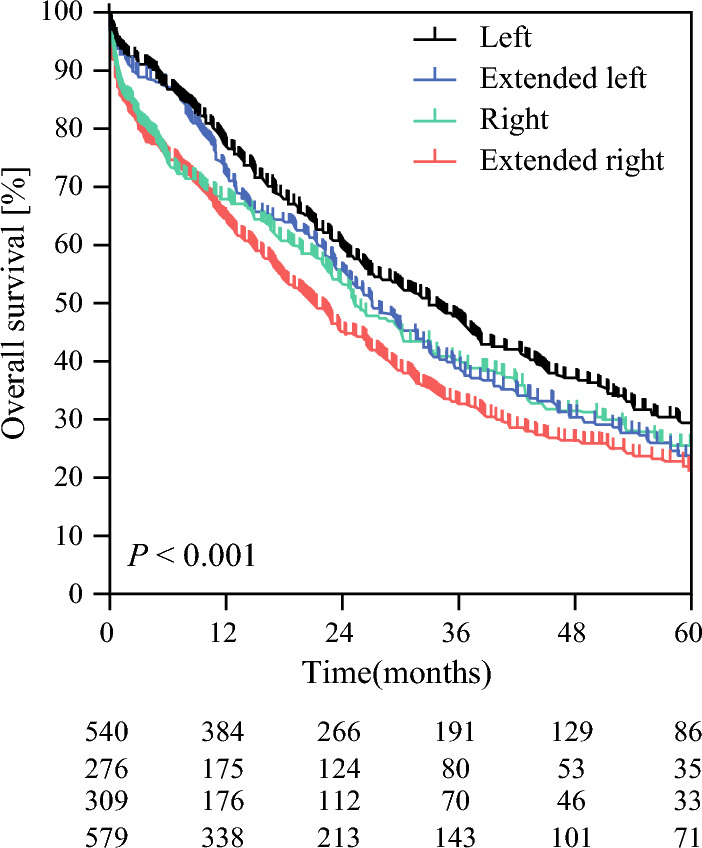
Overall survival of patients who underwent major liver resection for perihilar cholangiocarcinoma according to the type of hepatectomy

At last follow-up, 1145 patients had died, with a median follow-up of survivors of 63 months (95% CI 57–68). The median OS was 26 months (95% CI 25–28), with a 5-year OS of 25%. Median OS was 30 months (95% CI 27–34) after left and 23 months (95% CI 20–25) after right liver resection (*p* < 0.001). Survival curves for each type of hepatectomy are shown in Fig. 1. Median OS was 33 months (95% CI 28–38) after left, 27 months (95% CI 23–32) after extended left, 25 months (95% CI 21–30) after right, and 21 months (95% CI 18–24) after extended right hepatectomy (*p *< 0.001). The 1-year survival rates were 77% after left, 73% after extended left, 68% after right, and 65% after extended right hepatectomy. After exclusion of patients who died within 90 days after surgery, median OS was 37 months (95% CI 33–42) after left, 32 months (95% CI 27–36) after extended left, 33 months (95% CI 26–41) after right, and 28 months (95% CI 25–32) after extended right hepatectomy (*p* = 0.019).

After adjusting for other independent prognostic factors, the differences in survival between (extended) right and (extended) left hepatectomy remained (Table 3). Similar results for OS were found after excluding patients with 90-day postoperative mortality (Online Resource Table S2).

**Table 3:** Univariable and multivariable analysis for overall survival after resection of perihilar cholangiocarcinoma

	Univariable		Multivariable	
	HR (95% CI)	*p*-Value	HR (95% CI)	*p*-Value
Age	1.01 (1.00–1.01)	0.052	1.01 (1.00–1.01)	0.005
Male sex	1.17 (1.03–1.31)	0.012	1.17 (1.04–1.32)	0.011
ASA score III/IV vs. I/II	1.39 (1.24–1.57)	<0.001	1.25 (1.09–1.42)	0.001
Bismuth type				
I/II	Reference		Reference	
IIIA	1.17 (0.97–1.42)	0.102	0.91 (0.74–1.11)	0.358
IIIB	1.04 (0.86–1.27)	0.680	1.05 (0.85–1.31)	0.635
IV	1.34 (1.11–1.64)	0.003	1.03 (0.83–1.27)	0.789
Tumor size, >3 cm	1.22 (1.07–1.39)	0.003	1.12 (0.98–1.28)	0.103
Biliary drainage	1.21 (1.03–1.42)	0.020	1.01 (0.83–1.22)	0.957
Preoperative cholangitis	1.17 (1.01–1.36)	0.036	1.10 (0.95–1.29)	0.214
Type of hepatectomy				
Left	Reference		Reference	
Extended left	1.23 (1.03–1.47)	0.023	1.15 (0.95–1.39)	0.156
Right	1.23 (1.03–1.47)	0.021	1.39 (1.13–1.71)	0.002
Extended right	1.41 (1.22–1.63)	<0.001	1.46 (1.21–1.75)	<0.001
Portal vein resection	1.25 (1.11–1.42)	<0.001	1.08 (0.94–1.25)	0.260
Caudate lobe resection	0.99 (0.87–1.14)	0.973		
Node positive (pN+)	1.89 (1.67–2.13)	<0.001	1.68 (1.48–1.90)	<0.001
Metastatic disease	1.57 (1.19–2.07)	0.001	1.21 (0.90–1.61)	0.203
Positive margin	1.63 (1.44–1.84)	<0.001	1.43 (1.26–1.63)	<0.001
Poor differentiation	1.60 (1.38–1.85)	<0.001	1.35 (1.17–1.55)	<0.001
Perineural invasion	1.48 (1.26–1.73)	<0.001	1.26 (1.07–1.48)	0.005

*ASA* American society of anesthesiologists, *CI* Confidence interval, *HR* Hazard ratio

**Fig. 2:**
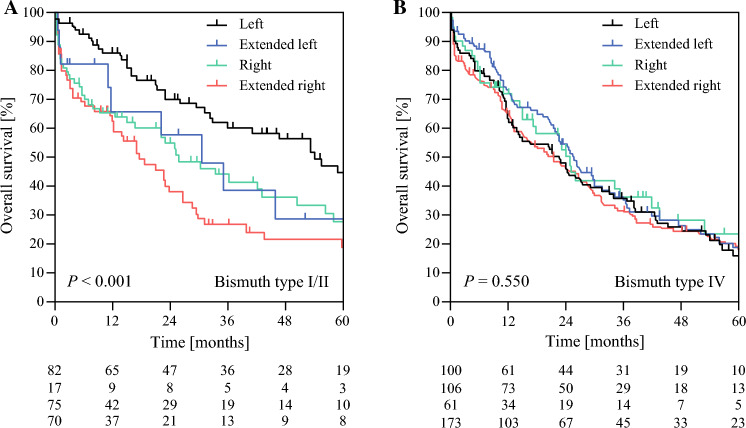
Overall survival after resection for Bismuth type **A** I/II and **B** IV perihilar cholangiocarcinoma according to the type of hepatectomy. Differences were tested using log-rank tests, and the numbers of patients at risk are shown below the graph

In the subgroup of Bismuth type I and II tumors, the median OS was 54 months (41–67) after left hepatectomy, compared with 30 months (12–49) after extended left, 26 months (14–17) after right, and 18 months (9–27) after extended right hepatectomy (*p* < 0.001) [Fig. 2a]. R0 margins were achieved in 81%, 53%, 75%, and 76% of left, extended left, right, and extended right hepatectomy, respectively (*p* = 0.122). For Bismuth type IV tumors, the median OS was 22 months (14–31) after left, 26 months (22–30) after extended left, 25 months (22–28) after right, and 21 months (14–28) after extended right hepatectomy (*p* = 0.824) [Fig. 2b]. R0 margins were achieved in 56%, 58%, 58%, 65% of left, extended left, right, and extended right hepatectomy, respectively (*p* = 0.160).

## Discussion

This study of 1701 patients who underwent major liver resection for pCCA showed that 90-day mortality was 9% after (extended) left hepatectomy and 18% after (extended) right hepatectomy (*p* < 0.001). OS was also better after (extended) left hepatectomy (30 months) compared with (extended) right liver resection (21 months). This difference remained present after adjusting for all relevant prognostic variables and remained after exclusion of patients who died within 90 days after surgery.

Several tumor characteristics can dictate the type of liver resection. Unilateral atrophy is observed in about one-third of patients and dictates ipsilateral resection.^7^ The extent of tumor mass towards the left or right liver (i.e. Bismuth 3A or 3B) may guide many surgeons to resecting the ipsilateral side (i.e., right-sided resection for 3A).^19^ Involvement of hilar vessels, most commonly the left portal vein or right hepatic artery, can also guide surgeons to resecting the ipsilateral side to avoid a vascular reconstruction.^10,20^ In patients without atrophy and without unilateral biliary tumor extension, both a left- and right-sided resection can be considered.

Numerous studies have investigated left versus right liver resection for patients with pCCA. All studies on this topic were retrospective and mostly single-center series from expert centers including fewer than 200 patients. Most studies reported similar perioperative and survival outcomes for patients who underwent a left or right liver resection.^8,21–25^ An Italian series of 124 patients reported a more than threefold higher postoperative mortality rate in right-sided resection,^9^ while a larger French study with 366 patients reported a twofold higher mortality in right-sided resections.^26^ The twofold difference in mortality is comparable with the current study.

The main downside of right-sided liver resections is the smaller remnant liver that predisposes patients to post-hepatectomy liver failure and mortality. In the literature, 90-day mortality rates are 1.5–4-fold higher after right- compared with left-sided liver resection for pCCA.^9,11,27^ Most deaths (about 90%) after surgery for pCCA are due to liver failure.^27^ On average, the right hemi-liver is twice as large as the left hemi-liver. After an extended right hemi-hepatectomy, only segments 2 and 3 remain. These two segments generally constitute only 5–27% of the total liver volume.^28^ In the presence of preoperative cholangitis (about 25% of patients), the risk of postoperative liver failure becomes even higher.^7,12,26,29^ Preoperative optimization with portal vein embolization (PVE) aims to increase the future liver remnant volume and function.^26,30^ PVE can result in a threefold reduction of liver failure in patients with pCCA.^7^ In the present study, PVE was only performed in 20% of right resections and 43% of extended right resections.

Long-term survival in this report was higher for patients who underwent (extended) left hepatectomy after adjusting for known independent prognostic factors. This was partially attributable to lower perioperative mortality after (extended) left hepatectomy, but OS remained superior after exclusion of patients with 90-day postoperative mortality. Previous single-center studies of fewer than 200 patients, and a meta-analysis including 1031 patients, did not find better survival for left-sided hepatectomies compared with other hepatectomies.^8,25,31^ The smaller sample size may explain why other studies did not find a difference in OS between left- and right-sided resections. An Italian series found a 10-month longer OS after right liver resections, which may also be attributed to the smaller sample size.^9^ This study is the largest to date on this topic and challenges the proposed oncologic superiority of extended right liver resection.^10^ The supposed higher R0 resection rate with extended right liver resection was not present in this real-world multicenter study.

This study has several limitations, which are mostly related to the retrospective study design, which is subject to selection bias. The retrospective, multicenter design resulted in differences in diagnostic work-up and treatment across centers. The criteria that centers used for a left- or right-sided resection were not recorded. However, the different approach of centers was also an advantage of this study. It allowed to compare similar patients who underwent different treatments (i.e., left- or right-sided resection). Although in many patients the decision to perform a left or right liver resection will be determined by tumor characteristics, the estimate of 90-day mortality and long-term OS remains relevant in the decision process and can guide the process for those patients in whom both left- and right-sided resection are feasible. Although a multivariable analysis was performed to correct for the decision to perform a certain type of liver resection, it could be that unrecorded factors still have guided the clinical decision to perform a left or right liver resection. For instance, the relatively low number of patients who underwent PVE before extended right liver resection could indicate right portal vein involvement with consequent left liver hypertrophy in some patients.

## Conclusion

This study showed that for patients with pCCA, a left hemihepatectomy is associated with less postoperative liver failure and 90-day mortality compared with right resections. Long-term prognosis was better for patients after left hemihepatectomy, even after the exclusion of all 90-day postoperative fatalities. When both a left and right liver resection is technically feasible in a patient with pCCA, a left-sided liver resection is preferred.

### Supplementary Information

Below is the link to the electronic supplementary material.

Supplementary file1 (DOCX 15 kb)
