# Supplementary material for: Higher Postoperative Mortality and Inferior Survival After Right-Sided Liver Resection for Perihilar Cholangiocarcinoma: Left-Sided Resection is Preferred When Possible
Source: Ann Surg Oncol. 2024 Mar 12;31(7):4405–12. doi: 10.1245/s10434-024-15115-0 (PMC11164810; doi:10.1245/s10434-024-15115-0)
Supplement: Supplementary file 1 — Supplementary file1 (DOCX 15 kb) [file 10434_2024_15115_MOESM1_ESM.docx]

**Table S1:** Patient and disease characteristics as well as outcomes according to the type of hepatectomy performed for pCCA divided into left and right-sided liver resection.

|  |  | |  |
| --- | --- | --- | --- |
|  | **Left-sided**  *n = 816* | **Right-sided**  *n = 885* | **P value** |
| **Age** | 66 (57-72) | 64 (56-72) | 0.034 |
| **Male sex** | 486 (60) | 495 (56) | 0.141 |
| **ASA score III/IV** | 282 (35) | 293 (33) | 0.459 |
| **Bismuth classification**  *I/II*  *IIIA*  *IIIB*  *IV* | 97 (12) 87 (11)  411 (51)  206 (26) | 145 (17)  453 (52)  37 (4)  234 (27) | < 0.001 |
| **Preoperative biliary drainage**  *None*  *PTBD*  *EBD*  *Both* | 178 (22) 208 (25) 334 (41) 96 (12) | 113 (13) 204 (23) 415 (47)  152 (17) | < 0.001 |
| **Preoperative cholangitis** | 155 (19) | 187 (21) | 0.272 |
| **Portal vein embolization** | 23 (3) | 308 (35) | < 0.001 |
| **Portal vein reconstruction** | 195 (24) | 340 (38) | < 0.001 |
| **Node positive** | 339 (42) | 378 (43) | 0.658 |
| **Metastatic disease** | 32 (4) | 42 (5) | 0.476 |
| **Negative margin** | 534 (65) | 585 (66) | 0.798 |
| **Poor differentiation** | 180 (22) | 210 (24) | 0.420 |
| **Perineural invasion** | 522 (64) | 611 (69) | 0.003 |
| **Major morbidity** | 334 (41) | 497 (56) | < 0.001 |
| **Liver failure***, ISGLS B/C* | 80 (10) | 194 (22) | < 0.001 |
| **Biliary Leakage***, ISGLS B/C* | 167 (20) | 172 (19) | 0.627 |
| **Hemorrhage***, ISGLS B/C* | 47 (6) | 65 (7) | 0.204 |
| **30-day mortality** | 47 (6) | 102 (12) | < 0.001 |
| **90-day mortality** | 73 (9) | 159 (18) | < 0.001 |

*Abbreviations: ASA: American Society of Anesthesiologists, PTBD: Percutaneous transhepatic biliary drainage, EBD: endoscopic biliary drainage, ISGLS: International study group of liver surgery.*

|  | **Univariable** | | **Multivariable** | |
| --- | --- | --- | --- | --- |
|  | **Hazard ratio (95%CI)** | **P value** | **Hazard ratio (95%CI)** | **P value** |
| **Age** | 0.99 (0.99-1.01) | 0.798 |  |  |
| **Male sex** | 1.16 (1.01-1.32) | 0.033 | 1.14 (1.00-1.30) | 0.059 |
| **ASA score** *III/IV versus I/II* | 1.30 (1.10-1.46) | 0.001 | 1.19 (1.03-1.38) | 0.022 |
| **Bismuth type**  *I/II*  *IIIA*  *IIIB*  *IV* | Reference 1.20 (0.96-1.51) 1.18 (0.94-1.48) 1.45 (1.16-1.82) | 0.105 0.146 0.001 | Reference 0.89 (0.71-1.13) 1.09 (0.86-1.40) 1.04 (0.82-1.32) | 0.344 0.474 0.762 |
| **Tumor size***, > 3 cm* | 1.24 (1.106-1.45) | 0.009 | 1.12 (0.94-1.33) | 0.215 |
| **Biliary drainage** | 1.18 (0.99-1.41) | 0.064 | 0.98 (0.79-1.21) | 0.818 |
| **Preoperative cholangitis** | 1.12 (0.94-1.32) | 0.196 | 1.04 (0.88-1.24) | 0.628 |
| **Type of hepatectomy**  *Left*  *Extended left*  *Right*  *Extended right* | Reference  1.24 (1.03-1.50) 1.10 (0.90-1.34) 1.27 (1.08-1.48) | 0.026 0.367 0.004 | Reference 1.20 (0.97-1.48) 1.32 (1.05-1.67) 1.35 (1.10-1.66) | 0.090 0.020 0.004 |
| **Portal vein resection** | 1.20 (1.04-1.38) | 0.010 | 1.01 (0.86-1.18) | 0.940 |
| **Caudate lobe resection** | 1.06 (1.91-1.24) | 0.455 |  |  |
| **Node positive** | 2.01 (1.76-2.30) | < 0.001 | 1.76 (1.52-2.02) | < 0.001 |
| **Metastatic disease** | 1.67 (1.25-2.23) | 0.001 | 1.20 (0.88-1.64) | 0.246 |
| **Positive margin** | 1.70 (1.48-1.94) | < 0.001 | 1.49 (1.30-1.72) | < 0.001 |
| **Poor differentiation** | 1.68 (1.44-1.96) | < 0.001 | 1.41 (1.21-1.65) | < 0.001 |
| **Perineural invasion** | 1.65 (1.38-1.97) | < 0.001 | 1.37 (1.14-1.64) | 0.001 |

**Table S2:** Uni- and multivariable analysis for survival after resection of perihilar cholangiocarcinoma after exclusion of patients who died within 90-days after surgery
